# Supplementary material for: The effect of sleep on public good contributions and punishment: Experimental evidence
Source: PLoS One. 2020 Oct 29;15(10):e0240324. doi: 10.1371/journal.pone.0240324 (PMC7595432; doi:10.1371/journal.pone.0240324)
Supplement: S1 Appendix — (DOCX) [file pone.0240324.s001.docx]

**Supplementary Appendix Fig 1: Testing characteristic differences across treatment assignment**

**Notes:** Coefficient estimates are from the binary regression of each characteristic on the SR indicator variable. Thinner (thicker) lines indicate the 95% (90%) confidence interval around the point estimates.
