# Supplementary material for: The effect of sleep on public good contributions and punishment: Experimental evidence
Source: PLoS One. 2020 Oct 29;15(10):e0240324. doi: 10.1371/journal.pone.0240324 (PMC7595432; doi:10.1371/journal.pone.0240324)
Supplement: S2 Appendix — (DOCX) [file pone.0240324.s002.docx]

**S1 Appendix. Experiment Instructions (screen shots of Veconlab instructions)**

**NO PUNISHMENT treatment**

**Instructions (ID = ), Page 1 of 6**

| - **Matchings:** The experiment consists of a series of **rounds**. In each round, you will be matched with the **same** group consisting of all 18 participants, i.e. you and 2 other people. The decisions that you and the other people make will determine the amounts earned by each of you. - **Investments:** You begin each round with a number of "tokens," which may either be kept or invested. The 2 people you are matched with will decide how many of their tokens to keep, and how many to invest. You will be not be able to see the others' decisions until after your decision is submitted. - **Earnings:** The payoff to you will equal: **$0.08** for each token you keep,  **$0.05** for each token you invest, and  **$0.05**for each token invested by the 2 other people who you are matched with. - **Subsequent Matchings:**You will be in the same group of 3 participants in all subsequent rounds, so the 2 other people you are matched with in one round are the same people that you are matched with in the next round.   Top of Form  Bottom of Form |
| --- |

**Instructions (ID = ), Page 2 of 6**

| **Example:** Suppose you have only two tokens for the round, and the earnings from tokens kept, invested, and invested by the others are **$0.10**, **$0.08**, and **$0.05** respectively.   - If you keep both tokens, then your earnings will be: **$0.10 x 2 = $0.20** from the tokens kept, plus **$0.05** times the number of tokens invested by the other people in your group. - If you invest both tokens, then your earnings will be: **$0.08 x 2 = $0.16** from the tokens invested, plus **$0.05** times the number of tokens invested by the other people in your group. - If you keep one and invest one, then your earnings will be: **$0.10 x 1 = $0.10** from the token kept, plus  **$0.08 x 1 = $0.08** for the token invested, plus  **$0.05** times the number of tokens invested by the other people in your group.   **Note:** In each of the 3 above cases, what you earn from the others' investments is: **$0.00** if the others invest 0 tokens, **$0.05** if the other people invest 1 token (in total) and keep the rest, **$0.10** if the other people invest 2 tokens (in total), etc.  Top of Form    Bottom of Form |
| --- |

**Instructions (ID = ), Page 3 of 6**

| - There will be **10 rounds**, and in all rounds you will begin with a new endowment of **15 tokens**, each of which can either be kept or invested. The 2 other people in your group will also have 15 tokens. - Everybody earns money in the same manner: **$0.10** for each token kept, **$0.08** for each token invested, and **$0.05** for each token invested by the 2 other people. - At the start of a new round, you will be given a new endowment of **15 tokens**. You are free to change the numbers of tokens kept and invested from round to round. - **Note:**You will be matched with the **same** people in all rounds.   Top of Form    Bottom of Form |
| --- |

### Instructions (ID = ), Page 4 of 6

| In the following examples, please select the best answer.  Earnings reminder: **$0.10** for each token you keep, **$0.08** for each token you invest, and **$0.05** for each token invested by others.  Top of Form  **Question 1:** Suppose you invest X of your 15 tokens and the total number invested by the 2 other people is Y tokens.  a) Then you earn (15 - X)*$0.10 + X*$0.08 .   b) Then your earnings will be at least as high as (15 - X)*$0.10 + X*$0.08.   **Question 2:** Which is true?  a) You may divide your 15 tokens any way you wish in each round, keeping some and investing some, or you may keep or invest them all.   b) The more you invest in one round the less there is to invest in later rounds.     Bottom of Form |
| --- |

**_________________________________________________________________________________**

### Instructions (ID = ), Page 5 of 6

| Top of Form  **Question 1:** Suppose you invest X of your 15 tokens and the total number invested by the 2 other people is Y tokens.  (a) Then you earn (15 - X)*$0.10 + X*$0.08 .   **(b) Then your earnings will be at least as high as (15 - X)*$0.10 + X*$0.08.**  Your answer, **(b)** is **Correct**, and how much more you earn depends on the **others' investments**.   **Question 2:** Which is true?  **(a) You may divide your 15 tokens any way you wish in each round, keeping some and investing some, or you may keep or invest them all.**   (b) The more you invest in one round the less there is to invest in later rounds.  Your answer, **(a)** is **Correct**; the only requirement is that the number kept and the number invested sum to 15 **in each round**.    Bottom of Form |
| --- |

**_________________________________________________________________________________**

**Instructions Summary (ID = )**

| - You will be matched with the **same** group of 2 other people in each round. There will be a total of **10 rounds** in this part of the experiment. - All people will begin with **15 tokens** which they may keep (and earn **$0.10** each) or invest (and earn **$0.08** each), knowing that they will also earn **$0.05** for each token invested by other people in the group. - You will begin each round with a new endowment of **15 tokens**, irrespective of how many tokens you may have kept or invested in previous rounds. - There will be a total of **10 rounds** in this part of the experiment. Your earnings for each round will be calculated for you and added to previous earnings, as will be shown in the total earnings column of the record form that you will see next.   Top of Form    Bottom of Form |
| --- |

**PUNISHMENT treatment**

**Instructions (ID = ), Page 1 of 6**

| - **Matchings:** The experiment consists of a series of **rounds**. In each round, you will be matched with the **same** group consisting of all 18 participants, i.e. you and 2 other people. The decisions that you and the other people make will determine the amounts earned by each of you. - **Investments:** You begin each round with a number of "tokens," which may either be kept or invested. The 2 people you are matched with will decide how many of their tokens to keep, and how many to invest. You will be not be able to see the others' decisions until after your decision is submitted. - **Earnings:** The payoff to you will equal: **$0.08** for each token you keep,  **$0.05** for each token you invest, and  **$0.05**for each token invested by the 2 other people who you are matched with. - **Punishments:** After seeing the others' decisions, you will be able to incur a cost to send "punishment points" that reduce the earnings of the recipients, as explained below. - **Subsequent Matchings:**You will be in the same group of 3 participants in all subsequent rounds, so the 2 other people you are matched with in one round are the same people that you are matched with in the next round.   Top of Form    Bottom of Form |
| --- |

**Instructions (ID = ), Page 2 of 6**

| **Example:** Suppose you have only two tokens for the round, and the earnings from tokens kept, invested, and invested by the others are **$0.08**, **$0.05**, and **$0.05** respectively.   - If you keep both tokens, then your earnings will be: **$0.08 x 2 = $0.16** from the tokens kept, plus **$0.05** times the number of tokens invested by the other people in your group. - If you invest both tokens, then your earnings will be: **$0.05 x 2 = $0.10** from the tokens invested, plus **$0.05** times the number of tokens invested by the other people in your group. - If you keep one and invest one, then your earnings will be: **$0.08 x 1 = $0.08** from the token kept, plus  **$0.05 x 1 = $0.05** for the token invested, plus  **$0.05** times the number of tokens invested by the other people in your group.   **Note:** In each of the 3 above cases, what you earn from the others' investments is: **$0.00** if the others invest 0 tokens, **$0.05** if the other people invest 1 token (in total) and keep the rest, **$0.10** if the other people invest 2 tokens (in total), etc.  Top of Form    Bottom of Form |
| --- |

**Instructions (ID = ), Page 2 (continued)**

| **Punishment Points**   \| **Punishment Points:** \| **0** \| **1** \| **2** \| **3** \| **4** \| **5** \| **6** \| **7** \| **8** \| **9** \| **10** \| \| --- \| --- \| --- \| --- \| --- \| --- \| --- \| --- \| --- \| --- \| --- \| --- \| \| **Receiver Cost:** \| **0** \| **0.1** \| **0.2** \| **0.3** \| **0.4** \| **0.5** \| **0.6** \| **0.7** \| **0.8** \| **0.9** \| **1** \| \| **Sender Cost:** \| **$0.00** \| **$0.10** \| **$0.20** \| **$0.30** \| **$0.40** \| **$0.50** \| **$0.60** \| **$0.70** \| **$0.80** \| **$0.90** \| **$1.00** \|  - **Punishment Points:** After investment decisions for all members of your group are revealed at the end of the round, you may send up to **10 punishment points**, which may directed to one or more others in your group. If you send no points, the cost to you is $0, and as you send more points, your cost increases, as shown in the bottom row of the table. The others can also send points to you, and their sending costs are determined in the same manner. - **Effect of Points Received:** Whoever receives points has their earnings for the round reduced by the relevant fraction shown in the second row of the table. For example, if you send 1 point to another person, their earnings will be reduced by a factor **0.1**, which is equivalent to multiplying their earnings by **0.9**. If that person receives 1 point from you and 1 point from another person, then their earnings reduction factor is given in the column for 2 points: **0.2**, etc. When you receive points, your earnings are reduced in the same manner. - **Earnings Adjustment:** If you send points, the cost of those sent points will be deducted from your earnings. If you receive points, your earnings (after costs have been deducted) will be reduced by a "tax" factor that depends on the total number of points received. - **No Anonymity:** The person receiving a punishment will see the sender's ID.   Top of Form    Bottom of Form |
| --- | --- | --- | --- | --- | --- | --- | --- | --- | --- | --- | --- | --- | --- | --- | --- | --- | --- | --- | --- | --- | --- | --- | --- | --- | --- | --- | --- | --- | --- | --- | --- | --- | --- | --- | --- | --- |

### Instructions (ID = ), Page 3 of 6

| - There will be **10 rounds**, and in all rounds you will begin with a new endowment of **15 tokens**, each of which can either be kept or invested. The 2 other people in your group will also have 15 tokens. - Everybody earns money in the same manner: **$0.08** for each token kept, **$0.05** for each token invested, and **$0.05** for each token invested by the 2 other people. In addition, costs of sending punishment points will be deducted, and your final earnings will then be reduced by a factor that depends on the total number of punishment points received. - At the start of a new round, you will be given a new endowment of **15 tokens**. You are free to change the numbers of tokens kept and invested from round to round. - **Note:**You will be matched with the **same** people in all rounds.   Top of Form    **Instructions (ID = ), Page 4 of 6**   \| In the following examples, please select the best answer, under the simplifying assumption that no punishments are sent or received.  Earnings reminder: **$0.08** for each token you keep, **$0.05** for each token you invest, and **$0.05** for each token invested by others.  Top of Form  **Question 1:** Suppose you invest X of your 15 tokens and the total number invested by the 2 other people is Y tokens.  a) Then you earn (15 - X)*$0.08 + X*$0.05 .   b) Then your earnings will be at least as high as (15 - X)*$0.08 + X*$0.05.   **Question 2:** Which is true?  a) You may divide your 15 tokens any way you wish in each round, keeping some and investing some, or you may keep or invest them all.   b) The more you invest in one round the less there is to invest in later rounds.     Bottom of Form \| \| --- \|   Bottom of Form |
| --- | --- |

**Instructions (ID = ), Page 5 of 6**

| Top of Form  **Question 1:** Suppose you invest X of your 15 tokens and the total number invested by the 2 other people is Y tokens.  (a) Then you earn (15 - X)*$0.08 + X*$0.05 .   **(b) Then your earnings will be at least as high as (15 - X)*$0.08 + X*$0.05.**  Your answer, **(b)** is **Correct**, and how much more you earn depends on the **others' investments**.   **Question 2:** Which is true?  **(a) You may divide your 15 tokens any way you wish in each round, keeping some and investing some, or you may keep or invest them all.**   (b) The more you invest in one round the less there is to invest in later rounds.  Your answer, **(a)** is **Correct**; the only requirement is that the number kept and the number invested sum to 15 **in each round**.    _Bottom of Form |
| --- |

### Instructions Summary (ID = )

| \| **Punishment Points:** \| **0** \| **1** \| **2** \| **3** \| **4** \| **5** \| **6** \| **7** \| **8** \| **9** \| **10** \| \| --- \| --- \| --- \| --- \| --- \| --- \| --- \| --- \| --- \| --- \| --- \| --- \| \| **Receiver Cost:** \| **0** \| **0.1** \| **0.2** \| **0.3** \| **0.4** \| **0.5** \| **0.6** \| **0.7** \| **0.8** \| **0.9** \| **1** \| \| **Sender Cost:** \| **$0.00** \| **$0.10** \| **$0.20** \| **$0.30** \| **$0.40** \| **$0.50** \| **$0.60** \| **$0.70** \| **$0.80** \| **$0.90** \| **$1.00** \|  - You will be matched with the **same** group of 2 other people in each round. There will be a total of **10 rounds** in this part of the experiment. - All people will begin with **15 tokens** which they may keep (and earn **$0.08** each) or invest (and earn **$0.05** each), knowing that they will also earn **$0.05** for each token invested by other people in the group. - In each round you may send up to 10 punishment points to one or more of the others in your group. The costs and earnings reduction ("tax") factors for points sent and received are shown in the table above. - The person receiving a punishment will see the ID number of the sender. - You will begin each round with a new endowment of **15 tokens**, irrespective of how many tokens you may have kept or invested in previous rounds. - There will be a total of **10 rounds** in this part of the experiment. Your earnings for each round will be calculated for you and added to previous earnings, as will be shown in the total earnings column of the record form that you will see next.   Top of Form    Bottom of Form |
| --- | --- | --- | --- | --- | --- | --- | --- | --- | --- | --- | --- | --- | --- | --- | --- | --- | --- | --- | --- | --- | --- | --- | --- | --- | --- | --- | --- | --- | --- | --- | --- | --- | --- | --- | --- | --- |
