# Supplementary material for: The effect of sleep on public good contributions and punishment: Experimental evidence
Source: PLoS One. 2020 Oct 29;15(10):e0240324. doi: 10.1371/journal.pone.0240324 (PMC7595432; doi:10.1371/journal.pone.0240324)
Supplement: Attachment — Submitted filename: Response to Reviewer 2 (PONE-D-20-04294R2).docx [file pone.0240324.s007.docx]

**Response to Reviewer #2 Comments: PONE-D-20-04294R1**

Thank you for detailed and thoughtful comments on the first revision of our manuscript. It appears that Review #1’s concerns were satisfied with our first revision, and so below we address only the points you have raised. Below we address your verbatim comments in order, explaining how we modified the manuscript to address the concerns raised, or explaining why we did not.

**Main comments and evaluation**

Motivation

“The manuscript lacks clear motivation both for why we should consider a mild 1 week sleep restriction treatment instead of a stronger intervention and why we should consider cooperation in a finitely repeated VCM game with and without punishment most relevant. These aspects should be made clear in the introduction and, concerning the VCM game, also in more detail in the design section (when discussing specific design elements).”

**RESPONSE:** We have modified the Introduction to more clearly identify the motivation for researching the effects of seemingly mild levels of sleep restriction over a one week period. We also support using this protocol with a new reference to Van Dongen et al, (2003) in the Introduction on page 1. Additionally, in our Design section on page 5 we highlight that our WR and SR treatment assignments were designed with the idea of comparing rested participants with participants with commonly experienced levels of insufficient sleep. Similarly, on page 2 of the Introduction we revised the text to highlight the relevance of studying VCM games with and without punishment (with a clarification added in the Design section on the bottom of page 6 highlighting the relevance and the point of a punishment-option treatment.)

-------------------------------------------------------------------------------------------------------------------

Selective attrition

“One important aspect of the study and its implications relates to potential selective attrition. The authors needed to exclude several participants in both conditions and discuss this fact only very briefly. However, it could well be that participants dropping out (particularly in the SR condition) bias the observed results. For instance, people complying with the SR condition may suffer less from sleep restrictions than those not complying or dropping out. Hence, differences in behavior may appear minor but would be much larger when such participants are included. I would at least expect a balance table that shows (by treatments) whether dropouts have similar characteristics.

Also I encourage the authors to discuss in the conclusion whether attrition may be a reason for why only minor effects are found. Note: In my view it is perfectly fine if no effect is detected, but the study does not provide a clear null result and appears to have power to identify only “intermediate” or “large”effect sizes. Being clearer about these aspects in the conclusion would make the paper even more transparent and help guiding future research on the topic.

Relatedly, the authors may provide an intention to treat analyses using treatment assignment (ignoring compliance) in the appendix (for those participants who finished the experiment).

Finally, when discussing characteristics of participants, it would be helpful to provide also results from balance tests in Table 1. E.g., there appears to be a higher variance in age in WR, a concern that may be alleviated by showing test results (that may also control for multiple hypotheses testing - but if so, the authors should apply such corrections everywhere).”

**RESPONSE:** We wish to thank the review for this cluster of comments, as the issue of selective attrition is an important one that we did not fully address previously. We explain below the several methods we use to do so.

First, we are fortunate that all of the participants who signed up for our specific study were drawn from a sleep survey database that has some measurable and demographic characteristics on potential participants. We were thus able to use these characteristics to address the selection issue as follows.

We first estimated a probit selection equation to predict the likelihood of the participant completing our protocol and VCM task, conditional on having signed up to participate in the first place (see Supplementary Appendix Table 2). Those who do not appear in our completed sample may have been lost due to being a no-show for the first Session of the experiment, or to mid-week protocol attrition. After predicting the probability of each subject “surviving” into out completed sample, we take its inverse (or “inverse probability weight”) and perform weighted regressions alongside our original non-weighted regressions. In other words, we used a standard *IPW*-correction so that individuals in our completed sample who shared measurable characteristics with those who were less likely to survive (based on the probit selection equation) are given more weight in the regressions (both the OLS and truncated Tobit). This approach is similar to that used in other literatures concerned with loss due to follow-up. We describe this methodology in detail in the last paragraph of page 9 (Results section).

While we run IPW selection corrections for every regression, for presentation purposes we report IPW models in Tables 4, 5, 6, and 7 as the ‘final’ treatment effect regression, and alongside any ‘interaction effect’ regression where the interaction of interest is found to be significant without IPW. (A new row towards the bottom of those tables indicates which models use IPW). We used this approach because there is not enough space to report a duplicated IPW version of every model without doubling the number of tables in a paper that is already full of results. We explain in text that we run IPW models in all cases, and believe this to be a reasonable approach given that the selection-corrected models do not change any of our key results.

The Reviewer second recommends an intent-to-treat analysis, but perhaps he/she did not see that we had already done this in our original paper in four Supplementary Appendix Tables for those participants who had completed the sleep protocol and VCM experiment but not complied. These estimations were our “compliant and noncompliant” sample analysis, which show qualitatively similar results compared to the “compliant-only” sample analysis presented in the main text. We now refer in the main text on page 9 to these Appendix Tables as intent-to-treat sample analysis.

Finally, as requested we have also added balance test results in Supplementary Appendix Figure 1 (also shown below). We find no statistically significant imbalance in age, gender, or CRT scores by treatment assignment (*p* > .10 in all instances). (Because of its bridge between our SR/WR sleep measure and *Personal SD*, we also compare the distribution of Optimal Sleep measures for SR vs. WR assigned subjects both before assignment (N=167), among those with complete data (N=126), and among those who are judged compliant (N=109), and find they never differ significantly.) As expected given the validity of our protocol, there is a statistically significant difference in ex post *Personal SD* by treatment assignment, was is demonstrated in Table 2 manipulation checks. We refer to this new Figure at the end of the 2^nd^ paragraph of the **Attrition and Compliance** subsection of the **Results** (page 8).

**Notes:** Coefficient estimates are from the binary regression of each characteristic on the SR indicator variable. Thinner (thicker) lines indicate the 95% (90%) confidence interval around the point estimates.

-------------------------------------------------------------------------------------------------------------------

Design features

“The design and different design elements need to be much better motivated. Open questions are:
• Why do the authors study a repeated game?
• Why is punishment designed as a second order public good?
• Why a partner and not a stranger matching?
• Why is punishment implemented as a reduction in points relative to points earned (e.g. 20 percent of the points another group member holds)? This results i) in stronger punishment for “the rich” at the same costs and ii) may add an additional layer of complexity on the game, as it becomes unclear how to coordinate on effective punishment. While the authors implemented a cap on how much points can be reduced, which almost never “bites”, it may still be that punishment decisions are affected by considerations about that cap and whether it might be reached (which may differ across WR/SR groups).
• Why is the id of punisher shown (i.e. why allow for counter punishment)?
• Why did you not inform people about the sleep conditions or assign them to pure WR/SR and mixed groups?

I ask the authors to explain and motivate these design choices more clearly and highlight why these elements are the most relevant when considering the effects of sleep restrictions. The authors may also use some of the questions above to discuss whether the weak effects observed may be a result of some of these choices.

Relatedly, it appears unclear from the manuscript whether participants were aware of both treatment conditions. Please explain in the manuscript whether this was the case and , if it was, why we should not worry about potential “contamination” effects. E.g. higher contributions of SR participants due to a willingness to signal to the experimenter that cooperation is stable even if one was sleep restricted.”

**RESPONSE:** We appreciate the reviewer’s concern over insufficient motivation and detail of our design details. Our design represented in part a choice we made to follow established VCM environments in order to position our results within the extant literature on VCM with/without punishment (e.g., punishment as 2^nd^ order public good, necessitating that we reveal the ID of individual contributors, and repeat interactions). Other features of design owed to the computerised platform we used (e.g., Veconlab with peer punishment features punishment points that reduce the recipient’s earnings by percentage rather than in absolute points, even though this yields a greater punishment for higher earners). We acknowledge that punishment in absolute rather than percentage earnings might have produced different results in our Conclusion as a limitation/caveat to our study (par. 2 page 28 ). However, our approach only “punishes” higher earners more if one considers punishment based on points levels. The issue of relative versus absolute punishment has been considered previously in:

Dickinson, D. L. (2001). The carrot vs. the stick in work team motivation. *Experimental Economics*, *4*(1), 107-124.

Based on this previous paper (which we mention here but do not cite in our current paper), it may be that this issue is most relevant in environments where endowments are heterogeneous across group members, which is not the case with our environment.

More generally, in that second paragraph of page 28 we now highlight that we aimed to mostly innovate via the sleep manipulation of the subjects and not the VCM environment itself for purposes of comparability, while recognizing that this may have affected our results. Similarly, we note that while a longer sleep manipulation could be considered, the one week at-home protocol has been shown powerful enough to produce important sleepiness and behavioral effects (citing two papers to support this claim at the end of that same 2^nd^ paragraph).

Finally, regarding the anonymity of participants’ sleep states, we now explain that we did this to better mimic real world applications where one does not know the sleep details of fellow group members (par. 2 page 7). From an experimental design standpoint, this feature gives us more confidence that any estimated effects are truly due to the sleep state and not due to the confound of beliefs that may be introduced (group member decisions in our environment are based only on observable outcomes in the repeated interaction environment, and not based on expectations of how sleepy or rested individuals should behave.)

-------------------------------------------------------------------------------------------------------------------

Analyses

“The way how non-parametric tests are applied appears not meaningful, as it ignores dependencies among group members and individuals. Why not report comparisons based on group level averages? Similarly, the regression analyses do not take into account that individual decisions are not only prone to within subject dependence but also depend on group characteristics (such that error terms may be correlated within groups). Why not use alternative specifications, e.g., individual random effects models with clustering on the group level?”

**RESPONSE:** With regards to our preliminary non-parametric analysis, we recognize that individual averaged decisions ignore how individuals are influenced by other group members, but cannot move the analysis to group level because groups had individuals of mixed sleep status. Explicitly acknowledging this limitation (par. 3 p. 14), we present our regression analysis as most pertinent. We now cluster standard errors to the group rather than individual level. While we view random effects or pooled cross section as equally valid models, our inverse-probability weight corrected models could not be run using random effects (as p weights cannot be used in STATA random effects), and so we retained pooled cross section. We did limited comparisons that found that, without IPW, random effects and pooled cross section yielded very similar results.

“Further, I consider it worthwhile to study counter punishment across treatment conditions as IDs were displayed.”

**RESPONSE:** We believe the reviewer may have missed that this was already in the paper, because while it was contained in our previous estimation results tables, we failed to note the counter-punishment main effect in our text, since we focused on its interaction effects with sleep status. We examine the issue of counter-punishment in Tables 6 and 7 with the variable *Lagged Punishment Received from Other* in models 4-8. Model 8 contains the interaction with sleep status. We now make note of this on par. 2 page 25. In our main analysis, the coefficient on this variable indicates that counter-punishment is always significant, but predominantly it does not differ by SR-WR assignment. There is an exception in the intent-to-treat estimation using the binary SR assignment, where there is a marginally significant increase in counter-punishment among SR individuals (Suppl. Appendix Table 5, models 8 and 9 interaction term *SR*Lagged Pun Received from Other*). However this effect is not found for the compliant sample with or without IPW correction, nor for any sample using the *Personal SD* sleep measure. As such, this effect is not robust and so we do not focus on it. Rather, in the text just before the *Conclusion*, we note that counter-punishment does not differ by SR status.

“Finally, it may be helpful to add a first round analyses in the appendix showing whether the differences observed occur also initially before learning about others behaviors (i.e. when punishment does not yet include potential counter punishment).”

**RESPONSE:** We understand the interest in uncontaminated initial round analysis, but given that we used a within-subject design with and without punishment, and alternating treatment orders between sessions, to capture individuals with no prior experience of their groups would leave us with a small sample (N=109 rather than N=1962) with which to make treatment effect comparisons. We also would not be able to include control variables relying on lags or round dummies found to be significant in multiple round analysis. We would then not know if any differences found were attributable to initial beliefs and tastes, vs. confusion, change in controls, or loss of sample size.

“It could also be worthwhile to discuss the robustness of the definition of the non-compliance zone in more detail and/or simply provide ITT estimates in the appendix.”

**RESPONSE:** As noted previously, we believe the reviewer may have missed that we included intent-to-treat analysis already in the Appendix. Thus, we see that are results are largely similar when considering a (somewhat arbitrary) compliance cutoff or when dropping the concern over compliance altogether. However, as explained in the text on pages 8-9, we clearly identify our compliance zone and mention that it is data-driven (see also our footnote xiv with citation on this).

-------------------------------------------------------------------------------------------------------------------

Implications and conclusion:

“1) In my reading, the main result is basically: If at all, there are weak effects of sleep restircitions on cooperation behavior in the game considered. As the result is not very clear cut and the study is powered only to identify medium to large effect sizes I advise the authors to avoid making too broad statements such as saying that their results provide no “evidence that recent patterns of reduced sleep are harming voluntary public good funding in the home, community, or workplace” (as they only show that effects are small it in a very specific setting and sample – related to my comments on potential selective attrition).

**RESPONSE**: We understand the Reviewer’s point and have tried to modify the text in this part of the conclusion to be clearer on the limitations to our findings. When summarizing our most consistently identified effect (other than ‘no effect’ on contributions when punishment is unavailable) we believe it is that the availability of punishment increases contributions among the “sleepy” (found in 3 of the 4 modelling and sample options across the main text and Appendix tables). This seems a fair conclusion given that this finding persists using *IPW* correction for sample selection. Unexpectedly, our move to clustering standard errors by group rather than individual also served to increase significance thresholds in our key specifications from 10% to 5%.

“2) The authors should also discuss more explicitly potential reasons for why the observed effects are small (among them: selective attrition, i.e. those who suffer most do not adhere to SR treatment and thus are excluded, hence they study reactions of people who may cope with sleep restrictions rather well as compared to the average person; exclusion of subjects with strong morning evening types, as they may be affected more / differently; power and identifiable effect size; specifics of the cooperation environment studied, e.g., mixed groups as compared to teams in which all members lack or do not lack sleep; second order public goods effects in punishment condition and complex beliefs about punishment and contribution of others; knowledge of both treatment conditions; etc.)”

**RESPONSE**: The Reviewer identifies a large plausible list of potential items that may have affected the size of our estimated sleep manipulation effects. However, given that this is an exploratory study, and that we initially explain (par.2 page 4) there are offsetting reasons *ex ante* why one might affect sleep restriction to increase *or* decrease contributions, and increase *or* decrease punishment, we do not feel the need to explain why the magnitude of results are what they are, instead of something else that we expected. Instead, our approach is to treat at length the issues we encountered that might threaten our ability to identify effects (attrition, compliance and power), and look for explanations for anomalies we encounter (e.g. divergence of SR/WR findings from personal sleep deprivation findings, even though optimal sleep distributions are balanced between SR/WR assigned subjects at the N=167, N=126 and N=109 stages.)

In the Conclusion (pp. 25-28) we thus discuss which modelling approaches we find more credible (as this may point to which sleep measure one should focus on). We try to be more explicit regarding the trade-offs faced in assessing our different specifications.

Beyond that, we think it appropriate simply to recognize that our results could have been different if we had taken different design decisions, as is true for every experiment. We have recognized that “more complex questions may only be answered with additional investigation using variations in protocol and cooperative dilemma environment.”

-------------------------------------------------------------------------------------------------------------------

Minor comments

“Page 1 Estimates of the economic costs of insufficient sleep appear very high to me. How reliable are they?”

**RESPONSE:** Published estimates regarding economic costs of sleep in the United States all immediately climb into the range of tens of billions of dollars. This is because they consider lost workplace productivity costs, generally estimated at 2% loss of productivity per “sleepy worker”, then multiply this by survey estimates that tens of millions of U.S. adults have insufficient sleep levels. Health costs in terms of ‘all-cause’ mortality are often then added in. See the Hafner et al.(2016) reference we cite, as they discuss their methodology in detail.

On Page 2 you say that “we primarily use binary random assignment (SR vs. WR) to treatment condition,” but the statement can be misleading. The authors study the effects of treatment on the treated and remain silent about potential selective attrition. That is, while treatment assignment is random, the analyses do not focus on intention to treat effects (i.e. comparing those randomly assigned with those not assigned to SR) but measure sleep status and comparing people adhering to the randomly assigned treatment conditions (SR/WR). The authors should thus be more precise here.

**RESPONSE**: Hopefully now that we have included *IPW-*corrected estimations to account for sample selection, and made clearer that we already included intent-to-treat analysis in the Appendix, this concern is now a moot point.

Page 3 You say “They found SR reduced subjects’ prosocial behaviors in general (including trust), which again may have implications for VCM outcomes” Be more precise and mention for which prosocial behaviors effects are found (and for which no effects are observed). This would help readers to put your findings in perspective.

**RESPONSE**: We now modify that text on page 4 to be more specific, which now reads “They found SR reduced subjects’ prosocial behaviors in general (including trust, trustworthiness, and altruistic giving)…”. Footnote iv was also amended to note that this Dickinson and McElroy study did not find sleep impacts on ultimatum bargaining. However, this is relegated to a footnote because it is an unclear result given the notorious difficulty of interpreting ultimatum game results that confound generosity and risk aversion (i.e. fear of zero payoff due to rejected offers).

Page 4 “…that sleep deprivation increases people’s aversion to exploitation (or suffering loss in social exchange) might” I think it is quite a stretch to claim that people feel exploited when being punished for not contributing.

**RESPONSE:** We were perhaps unclear here in our writing, but were referring to people who contributed more feeling exploited by others. We have moved this sentence down below the sentence in that same paragraph that notes the lack of SR studies involving punishment. In its place, we now simply note that “….any increased aversion to punishment may predict increased contributions under SR.”

Page 6 “allocated between” sounds unclear to me. Participants could decide on how many punishment points to assign to each other group member, right? PLease clarify in manuscript.

**RESPONSE**: This is now the final par. of page 7. We have reworded this to make it clearer. Yes, they could decide how many points to assign to each other member separately.

Page 8 "However, we could not conduct power analysis for such regression models to identify exact thresholds." You may potentially apply simulation based methods, see for example <https://link.springer.com/article/10.1007/s40881-016-0028-4> or <https://drive.google.com/file/d/1Neyb97eetVcFD842dUW_kgg_8DrOar6s/view>

**RESPONSE:** Upon some initial investigation of what would be involved, we appreciate the suggested simulation approach, but feel that this goes beyond the scope of our paper. Our goal was never to identify that we can detect an effect of the exact size *d*=.471 with power = .80 at *α* = .05. We are comfortable noting that our study is likely not sufficiently powered to identify effect sizes below the Cohen “medium” range (nor should that be a condition on experimental designs as it seems extreme to require that studies with costly set up always be sufficiently powered to even detect *small* effect sizes).

Page 10: “As foreshadowed, the variable Personal SD has use beyond manipulation checks, as an alternative continuous measure of inadequate sleep (personalized to perceived sleep need). In what follows we shall report results using both approaches. ” The variable “treatment impact” does so as well. It appears unclear why it is ignored here.

**RESPONSE:** While we did elicit other measures of perceived sleepiness to use as manipulation checks, including Treatment Impact and Karolinska Sleepiness, we viewed them as less credible than Personal SD as stand-alone measures of sleep status because only the latter is at least partially anchored to objective sleep scores. We refer to this in par. 2 on page 12. We also explain in the introduction that previous research has found partial sleep restriction causes decrements in people’s cognitive functioning before they are aware of greater sleepiness, suggesting people’s self-awareness regarding sleep restriction effects is imperfect.

Page 10 “While the dominant strategy remained complete free-riding and efficiency full contribution, the exact MPCR differed, as did the complexity of the incentives. For preliminary non-parametric tests, we thus present results with and without cohort 1 (since the exchange rate change does not affect relative incentives), and test whether or not cohort 3 can be pooled with the other cohorts.” The statement in “()” is only true for selfish decision makers, e.g., inequality averse decision makers tradeoffs could change.

**RESPONSE:** We agree with the reviewer that different preferences make such statements more qualified. Since we intended to refer to the typical “money-maximizer” preferences, we have added “(money maximizing)” in parenthesis to make this clearer.

Appendix

“Figures in the pdf are not readable (downloaded figures are ok).”

**RESPONSE:** We apologize. In our last submission, we were already asked to put the revised paper in full format for PLOS ONE publication. The TIF figures we uploaded using the website’s dedicated portal for acceptable final form are indeed too small. We shall seek to upload larger images in a still acceptable format through the portal.
